# Differentiation of human colon tissue in culture: Effects of calcium on trans-epithelial electrical resistance and tissue cohesive properties

**DOI:** 10.1371/journal.pone.0222058

**Published:** 2020-03-05

**Authors:** Shannon D. McClintock, Durga Attili, Michael K. Dame, Aliah Richter, Sabrina S. Silvestri, Maliha M. Berner, Margaret S. Bohm, Kateryna Karpoff, Caroline L. McCarthy, Jason R. Spence, James Varani, Muhammad N. Aslam

**Affiliations:** 1 Department of Pathology, The University of Michigan Medical School, Ann Arbor, Michigan, United States of America; 2 Department of Cell & Developmental Biology, The University of Michigan Medical School, Ann Arbor, Michigan, United States of America; 3 Department of Internal Medicine (The Division of Gastroenterology), The University of Michigan Medical School, Ann Arbor, Michigan, United States of America; Emory University School of Medicine, UNITED STATES

## Abstract

**Background and aims:**

Human colonoid cultures maintained under low-calcium (0.25 mM) conditions undergo differentiation spontaneously and, concomitantly, express a high level of tight junction proteins, but not desmosomal proteins. When calcium is included to a final concentration of 1.5–3.0 mM (provided either as a single agent or as a combination of calcium and additional minerals), there is little change in tight junction protein expression but a strong up-regulation of desmosomal proteins and an increase in desmosome formation. The aim of this study was to assess the functional consequences of calcium-mediated differences in barrier protein expression.

**Methods:**

Human colonoid-derived epithelial cells were interrogated in transwell culture under low- or high-calcium conditions for monolayer integrity and ion permeability by measuring trans-epithelial electrical resistance (TEER) across the confluent monolayer. Colonoid cohesiveness was assessed in parallel.

**Results:**

TEER values were high in the low-calcium environment but increased in response to calcium. In addition, colonoid cohesiveness increased substantially with calcium supplementation. In both assays, the response to multi-mineral intervention was greater than the response to calcium alone. Consistent with these findings, several components of tight junctions were expressed at 0.25 mM calcium but these did not increase substantially with supplementation. Cadherin-17 and desmoglein-2, in contrast, were weakly-expressed under low calcium conditions but increased with intervention.

**Conclusions:**

These findings indicate that low ambient calcium levels are sufficient to support the formation of a permeability barrier in the colonic epithelium. Higher calcium levels promote tissue cohesion and enhance barrier function. These findings may help explain how an adequate calcium intake contributes to colonic health by improving barrier function, even though there is little change in colonic histological features over a wide range of calcium intake levels.

## Introduction

An intact colonic barrier is necessary for gastrointestinal health [1–6]. The intact barrier prevents permeation of toxins, soluble antigens, lipopolysaccharides and other inflammatory initiators across the intestinal wall, while inhibiting infiltration of bacteria and other particulate matter into the interstitium. Intestinal barrier defects are seen in conjunction with inflammation in the gastrointestinal tract. While inflammation itself can give rise to these defects, pre-existing weaknesses in the gastrointestinal barrier could predispose the tissue to inflammation.

Multiple cell surface structures contribute to an effective colonic barrier. Most attention has been focused on tight junctions as these structures are found at the apical surface of the mucosal epithelium and form a “seal” between adjacent cells [7,8]. Along the lateral surface (i.e., beneath the apical surface) are desmosomes [9,10], which provide anchoring sites for intermediate filaments and are necessary for cohesive strength. In addition to these two cell-cell adhesional complexes are the adherens junctions [11–13]. These junctional complexes are comprised of cadherin family members and mediate homotypic cell-cell attachment. Their formation between adjacent epithelial cells is a rapid event and thought to be necessary for the establishment and organization of other junctional complexes. They may also contribute to cohesive strength directly or indirectly. In addition to cell-cell adhesion molecules, the basement membrane and moieties that mediate interactions between cells and the basement membrane also contribute to tissue barrier properties–especially in regard to cell trafficking and macromolecule permeability [14,15].

Calcium is critical to cell-cell and cell-matrix adhesion. Most of the molecules that mediate cellular adhesive functions depend on precise levels of calcium for homotypic and heterotypic interaction [16–18]. Equally important, calcium is critical to epithelial cell differentiation [19] and many of the proteins that make up cellular adhesion complexes are up-regulated as part of the differentiation response.

In recent studies, we established colonoid cultures from histologically-normal human colon tissue as well as from several large adenomas obtained at endoscopy [20,21]. Once established, the colonoid cultures were maintained in a low-calcium (0.25 mM) environment or exposed to levels of extracellular calcium (1.5–3.0 mM) that are known to foster epithelial cell differentiation. Calcium was provided either alone or as part of Aquamin, a multi-mineral natural product, that also contains a high level of magnesium and detectable amounts of up to 72 additional trace elements in addition to calcium [22]. To summarize findings from these studies, tumor-derived colonoids maintained an undifferentiated phenotype in low-calcium conditions but underwent a robust differentiation response with calcium supplementation. In contrast, normal tissue colonoids underwent differentiation in the low-calcium environment and additional calcium had only incremental effects on morphological features and differentiation marker (CK20) expression. As part of our study with histologically-normal colonoids, a combination of proteomic analysis and immunohistology was used to assess the effects of calcium intervention on cellular adhesion molecule expression. In parallel, transmission electron microscopy was used to visualize adhesion structures. Treatment with either calcium alone or Aquamin had only modest effects on tight junction protein expression but substantially increased desmosomal proteins and cadherin family members. Basement membrane proteins were also up-regulated. Adhesion structures, especially desmosomes, were prominent in the treated colonoids. Calcium from either source was comparably effective in stimulating cell-cell adhesion proteins, but Aquamin was more effective than calcium alone at up-regulating basement membrane molecules [21].

Given the changes in adhesion protein expression, we postulated that improved barrier function and, especially, stronger tissue cohesion would be consequences of intervention. However, no data were provided to substantiate this suggestion. The present study was carried out to investigate this hypothesis. Here we show that trans-epithelial electrical resistance (TEER), a measure of barrier integrity and permeability [23], was increased with calcium alone or with Aquamin supplementation but, consistent with the modest change in tight junction proteins expression, the increase was slight. In contrast, tissue cohesion was increased substantially in response to the same interventions. This was accompanied by a strong up-regulation of desmoglein-2 and cadherin-17.

## Materials and methods

### Calcium sources

Calcium Chloride was obtained as a 0.5 M solution (PromoCell GmbH, Heidelberg, Germany). Aquamin, a calcium-rich and magnesium-rich multi-mineral product obtained from the skeletal remains of the red marine algae, *Lithothamnion sp* [22] was provided by Marigot Ltd (Cork, Ireland) as a powder and has been used in previous studies [20,21,24,25]. Aquamin contains calcium and magnesium in a ratio of approximately 12:1, along with measurable levels of 72 other trace minerals (essentially all of the minerals algae fronds accumulate from the ocean water). Mineral composition was established via an independent laboratory (Advanced Laboratories; Salt Lake City, Utah) using Inductively Coupled Plasma—Optical Emission Spectrometry (*ICP***-***OES*). Aquamin is sold as a dietary supplement (GRAS 000028) and is used in various products for human consumption in Europe, Asia, Australia, and North America. A single batch of Aquamin^®^ Soluble was used for this study. The complete minerals composition can be found in S1 Table.

### Colonoid culture

Histologically normal colon tissue in colonoid culture was available from four subjects of our previous studies [20, 21]. The collection and use of human colonic tissue was approved by the Institutional Review Board (IRBMED) at the University of Michigan. This study was conducted according to the principles stated in the Declaration of Helsinki. All subjects provided written informed consent prior to flexible sigmoidoscopy. For the present study, cryopreserved samples were re-established in culture and expanded over a 3–4 week period as described by Dame et al. [26]. Briefly, biopsies were finely minced on ice using a #21 scalpel and seeded into Matrigel (Corning), prepared to 8 mg/ml. During the expansion phase, colonoids were incubated in growth medium. Growth medium consisted of 50% L-WRN [27] -conditioned Advanced DMEM/F12 (Invitrogen) providing a source of Wnt3a, R-spondin-3, and Noggin and was supplemented with 1X N2 (Invitrogen), 1X B-27 without vitamin A (Invitrogen), 1 mM N-Acetyl-L-cysteine, 10 mM HEPES (Invitrogen), 2 mM Glutamax (Invitrogen),10 μM Y27632 (Tocris), 500 nM A83-01 (Tocris), 10 μM SB202190 (Sigma), 100 μg/mL Primocin (InvivoGen), and 100 ng/mL EGF (R&D). In addition, 2.5 μM CHIR99021 (Tocris) was included for two days at each passage.

### TEER assay

One day prior to cultivation on transwell membranes, the colonoids were treated with a 1:1 mix of growth medium and IntestiCult-Human culture medium (StemCell Technologies). Colonoids were then dissociated into small cell aggregates (under 40μm in size) with 0.05% Trypsin-EDTA (Invitrogen) containing 10 μM Y27632 (3.75 minutes at 37°C) and plated onto collagen IV (Sigma)-coated transwells (0.4 μm pore size, 0.33cm^2^, PET, Costar) at 200,000 individual aggregates per well [28–30] in 24-well plate (Corning Costar). Cells were seeded for attachment and initial growth in growth medium. After 24 hours, the growth medium was replaced with KGM-Gold (Lonza)–a serum-free, calcium-free medium designed for epithelial cell growth, supplemented with calcium alone or with Aquamin to bring a final calcium concentration to 0.25, 1.5 or 3.0 mM was used for comparison. KGM-Gold containing 0.25 mM calcium was used for control.

As a positive control for monolayer integrity, Complete Differentiation Medium was included with each experiment. Complete Differentiation Medium has Advanced DMEM/ F12 (supplemented with AlbuMax–bovine serum albumin) media containing 1.05 mM calcium as its base but lacks the stem cell support components provided in growth medium [30]. Both the experimental media and Completed Differentiation Medium were supplemented with 10 nM Gastrin (Sigma), 50 ng/mL Noggin (R&D), 50 ng/mL EGF, and 2.5 μM Y27632. After 2 days in Complete Differentiation Medium or in the experimental formulations, Y27632 was removed. Media were refreshed every two days during the assay period. TEER values were determined on days-2 and -5 with an Epithelial Volt ohm meter 2 (EVOM2) and STX2 series chopstick electrodes (World Precision Instruments).

### Confocal fluorescence microscopy

At the completion of the TEER assay, membranes were prepared for confocal fluorescence microscopy. The membranes were fixed for 15 minutes at -20°C in methanol. They were then washed three times in PBS before blocking in 3% BSA (A8806; Sigma) in PBS for 1 hour. Following this, membranes were stained with antibodies to occludin, claudin-4, desmoglein-2 and cadherin-17 for 1 hour in 1% BSA in PBS. Antibody source and characteristics are presented in S2 Table. Stained membranes were rinsed 3 times (5 minutes each) in PBS, exposed to DAPI (D9564; Sigma-Aldrich) for 5 minutes to identify nuclei and washed an additional 3 times with PBS. Finally, the membranes were gently cut from the transwell insert and mounted apical side up on Superfrost Plus glass slides (Fisher Scientific, Pittsburgh, PA) with Prolong Gold (P36930; Life Technologies Molecular Probes). The stained specimens were visualized and imaged with a Leica Inverted SP5X Confocal Microscope System (University of Michigan Medical School Biomedical Research Core Facility). Confocal generated Z-stacks were rendered as a 3D movie using Fiji (ImageJ1.52n with Bio-Formats Importer plugin).

### Western blotting

At the completion of the TEER assay on day-5, transwell membranes (three per condition) were harvested for protein. Briefly, wells were washed gently with PBS 3 times, then protein was extracted using RIPA buffer (89901; Thermo Scientific). Monolayers were lysed by repetitive pipetting then incubating for 10 minutes on ice. Non-soluble cellular debris was removed by centrifugation at 14,000g for 10 minutes and protein was quantified using a BCA assay (23227; Pierce).

Samples were heated for 10 minutes at 70°C in NuPage LDS sample buffer and then run on 4–12% Bis-Tris gels using NuPage MOPS running buffer under reducing conditions. Proteins were then transferred onto nitrocellulose membranes, blocked with 5% non-fat dry milk and probed with the primary and appropriate secondary antibodies as shown in S2 Table. Secondary antibodies were used at 1:5000 for all membranes. β-actin was used as a loading control in each assay. SuperSignal WestPico Plus (34577; Thermo Scientific) detection reagent was used and bands were visualized by exposing the membranes on CL-XPosure Film (34090; Thermo Scientific). Some nitrocellulose membranes were reprobed using Restore Western Blot Stripping buffer (21059; Thermo Scientific). Relative band density was determined using ImageJ (1.52n) gel analysis tools. The original images of these western blots are presented in S1 File.

### Tissue cohesion assay

After establishment and expansion in colonoid culture as above, colonoids were incubated for a two-week period in L-WRN conditioned Advanced DMEM/F12 diluted 1:4 with KGM Gold. The final serum concentration in the medium was 2.5% and the calcium concentration was 0.25 mM. Since calcium is present as a component of the L-WRN conditioned medium, Aquamin could not be used in this assay at 0.25 mM calcium concentration. In parallel, colonoids were incubated in the same medium supplemented with calcium (alone or as part of Aquamin) to a final calcium concentration of 1.5 or 3.0 mM. At the end of the two-week incubation period, phase-contrast microscopy (Hoffman Modulation Contrast—Olympus IX70 with a DP71 digital camera) was used to capture images in order to measure the size of multiple individual colonoids (80–140 per condition). Colonoids were then separated from the Matrigel, fragmented with mechanical force alone by pipetting the entire pellet 30x through an uncut 200 microliter pipet tip. The fragments were washed 3x in PBS, and then re-cultured in fresh Matrigel. One day later, multiple colonoids were again examined under phase-contrast microscopy and sized. Phase-contrast images were analyzed using area measurements in Adobe Photoshop (CC version 19.1.5). Average colonoid size reduction was determined by dividing the average pre-fragmentation surface area by the average post-fragmentation area.

### Statistical analysis

Means and standard deviations were obtained for discrete values in both assays. Data generated in this way were analyzed by ANOVA followed by unpaired t-test (two-tailed) for comparison using GraphPad Prism version 8.

## Results

### Barrier integrity (measured by TEER)

**Fig 1:**
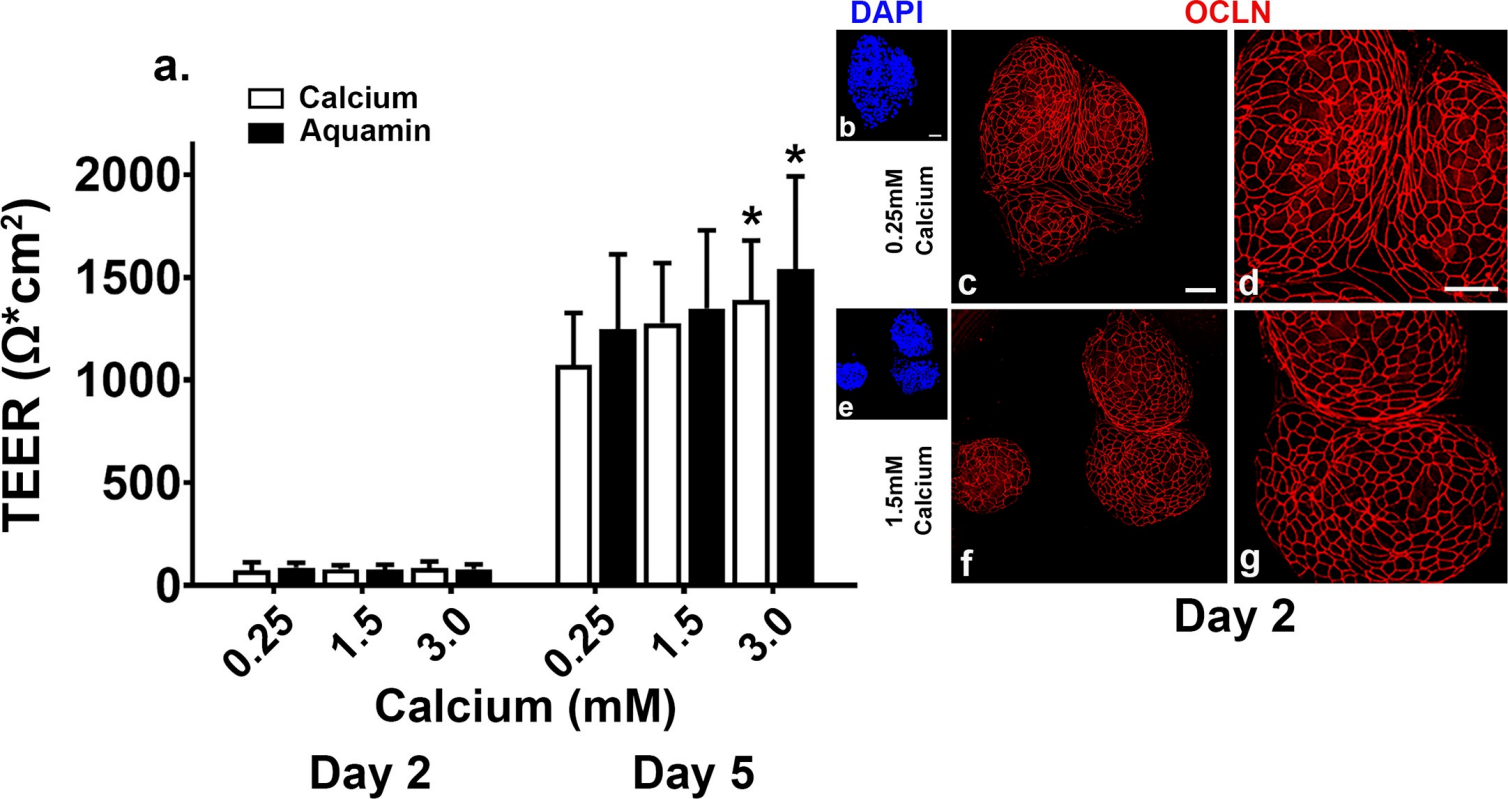
Trans-epithelial electrical resistance (TEER) values. Colonoids were plated on transwell membranes and incubated under the indicated conditions. **a**: At day-2 and -5, electrical resistance across the cell layer was assessed. Values shown are means and standard deviations based on five separate experiments with 3 or 4 samples (individual membranes) per data point in each experiment). Membrane to membrane variability was routinely less than 5%. Data were analyzed for statistical differences using ANOVA followed by unpaired-group comparisons. * indicate statistical significance from calcium at 0.25 mM (*p*<0.05; calculated by two-tailed unpaired t-test). **Inset**: Confocal fluorescent microscopic (max projected) images of membranes stained after the day-2 reading with DAPI (panels: **b,e**) or with antibody to occludin (panels: **c,d,f,g**). Scale bar = 10 μm. OCLN = occludin.

Fig 1 demonstrates TEER values obtained with colonoid cells at day-2 and day-5 from five independent assays. At the early time-point, resistance values were low under all experimental conditions, though TEER values were already high (1744 ± 744 ohms/cm^2^) in Complete Differentiation Medium (positive control). By day-5, substantial electrical resistance across the cell layer was seen under all experimental conditions. Values ranged between 59% of the maximum value (i.e., 1812 ± 15 ohms/cm^2^ in Complete Differentiation Medium) with 0.25 mM calcium and 85% of the maximum value with Aquamin providing 3.0 mM calcium. When Aquamin-treated colonoid cells were compared to colonoid cells treated with calcium alone at comparable calcium levels, Aquamin values were 16% higher than calcium alone at 0.25 mM, 6% higher at 1.5 mM, and 11% higher at 3.0 mM. The TEER values from 3.0 mM calcium and Aquamin were significantly higher as compared to the control (0.25 mM Calcium) with a *p* value of 0.043 and 0.015 respectively.

At the completion of the electrical resistance measurements on day-2 and day-5, membranes were fixed and stained in the transwell inserts. The day-2 membranes were stained with an antibody to occludin and DAPI (as a way to identify areas of the membrane that were devoid of cells) and the day-5 membranes were stained with the combination of antibodies to occludin and desmoglein-2 (single membrane). The inset to Fig 1 shows occludin staining at day-2. It is evident from the images (low- and high-calcium), that in either condition, large portions of the membrane (i.e., black areas) were devoid of cells. This is confirmed by the DAPI (nuclear staining) images shown as part of the inset. Thus, the lack of electrical resistance at day-2 reflects the fact that a monolayer of cells had not yet formed from the attached cell clusters. It is also evident from the high-power views that where cell clusters were deposited on the membranes, occludin staining was strong at 0.25 mM calcium and not perceptibly different with calcium at 1.5 mM.

**Fig 2:**
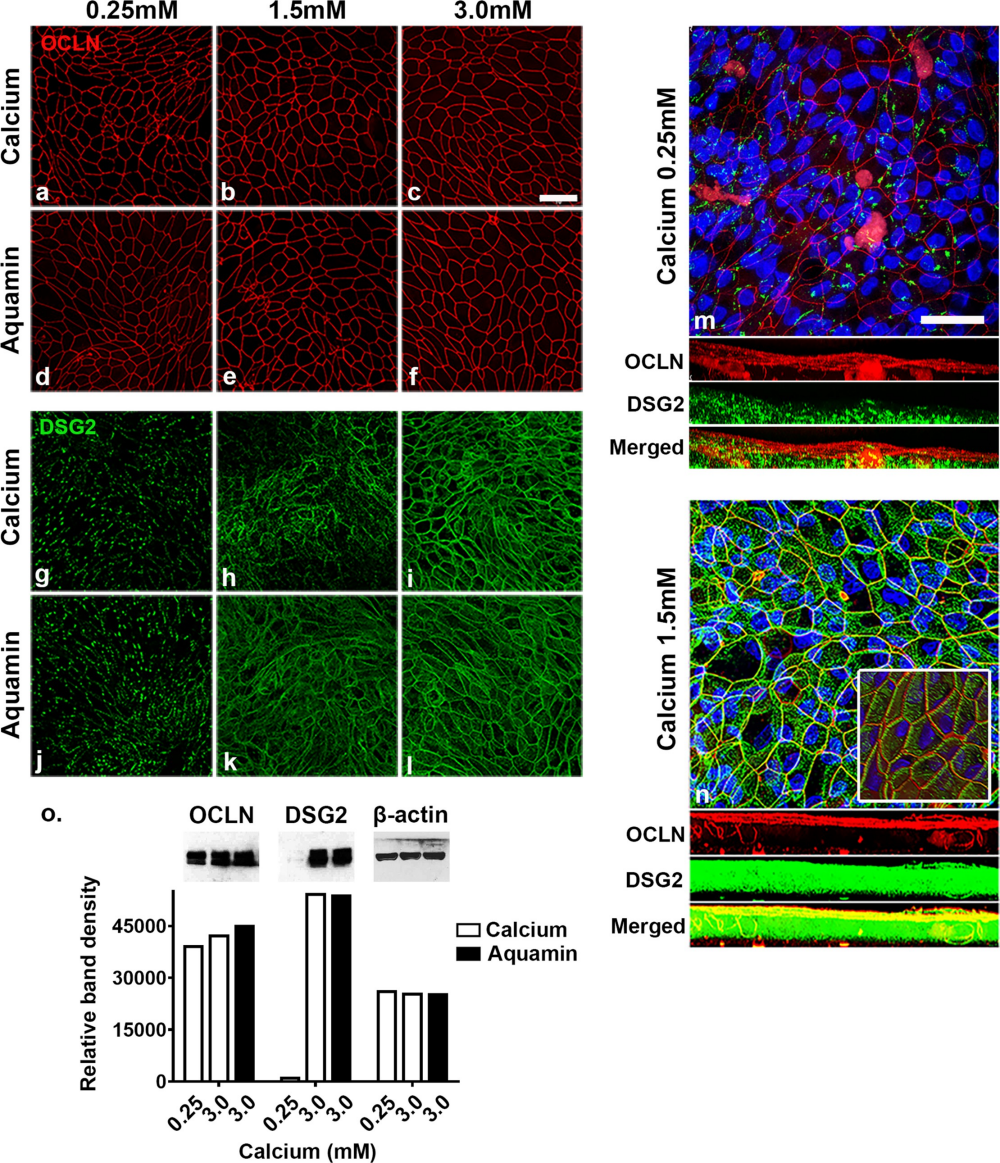
Confocal fluorescent microscopic images and western blot results at day-5. Colonoids were plated on transwell membranes and incubated under the indicated conditions. After TEER assessment at day-5, membranes were prepared and stained. **a-f**: Occludin (max projected); **g-l**: Desmoglein-2 (max projected). **m-n**: Occludin and desmoglein-2 (z-stack composites made up of approximately 50 planes per image). Nuclear staining is indicated by blue color (DAPI). The three bands below the main images represent horizontal views of staining through the cell layer at each z-plane. Occludin is red, desmoglein-2 is green and the bright yellow color in the horizontal view of the 1.5 mM calcium image represents a composite of the two proteins. **Inset**: The inset in the 1.5 mM calcium image is a view of the entire z-stack viewed from a 45^o^ angle to the cell surface in order to provide a 3-dimensional rendering. Scale bar = 10 μm. **o**: Western blot for occludin and desmoglein-2. 10 μg of protein from each condition was used. β-actin was used as a loading control. Band quantitation was done using ImageJ software. OCLN = occludin; DSG2 = desmoglein-2.

Day-5 staining results with antibodies to occludin and desmoglein-2 are shown in Fig 2. Panels (Fig 2A–2F) present findings with occludin. By day-5, an intact staining boundary could be seen between virtually all cells under all conditions. The staining intensity did not vary with the condition. The staining pattern at day-5 was indistinguishable from that at day-2. The staining pattern with desmoglein-2 was very different (panels g-l in Fig 2). There was detectable staining with both calcium alone and Aquamin at 0.25 mM. In both cases, however, staining was patchy; no intact staining boundary was detectable between cells. At 1.5 mM, strong staining was observed throughout the monolayer and an intact boundary between adjacent cells was evident. Though differences between the two interventions were generally small, a few areas of the membrane from the calcium-alone treatment continued to demonstrate patchy staining. At 3.0 mM, strong and uniform staining was seen throughout the cell layer with both interventions.

Panels m and n in Fig 2 show staining at 0.25 mM calcium and at 1.5 mM calcium with antibodies to both occludin and desmoglein-2. For this image, the entire “z-plane” stack is shown. The three broad bands shown below each image represent z-planes (approximately 50 individual planes) viewed horizontally through the cell layer from top to bottom. Under both low- and high-calcium conditions, virtually all of the red staining (occludin) is in the upper-most planes. There is little difference between the two calcium levels. With desmoglein-2 (green staining), calcium concentration-related differences are evident. At 0.25 mM calcium, most of the staining is in the lower planes while at 1.5 mM calcium, staining is distributed through the entire cell layer. The lowest band presents a merger of the two colors. It appears from the bright yellow color at 1.5 mM calcium that both proteins are present at the cell surface. Desmoglein-2 has been shown to cycle through the apical surface during barrier formation [31], and it is possible that the two proteins are, in fact, co-localized at the apical surface. Alternatively, the monolayer is not a geometrically-flat surface and some of the upper-most z-planes may include both apical surface and sub-surface views from multiple individual cells.

The inset to the high-calcium image helps to resolve this issue. It is a high-power view of the same stack of z-planes viewed at a 45-degree angle to provide a 3-dimensional (3D) representation. With occludin (red stain), the cell-cell boundary can be seen at the apical surface of the cells over the entire cell layer. desmoglein-2 (green stain) is seen at the surface but immediately below occludin, and extends downward along the lateral boundary between adjacent cells. The rotating 3D movie (S1 Movie) from confocal-generated Z-stacks (from high-calcium) also highlights the fact that occludin (red staining) is apical and desmoglein-2 (green staining) starts apically and extends laterally to cover the entire length of cells.

Panel o presents results from western blotting studies with the same two proteins. With occludin, band densities were similar, irrespective of calcium concentration. In contrast, desmoglein-2 expression was almost non-detectable in the low-calcium environment but strongly expressed at 3.0 mM calcium. Consistent with the results from confocal fluorescence microscopy, there were no detectable differences between calcium provided as a single mineral at this level and the combination of calcium and other minerals in Aquamin.

**Fig 3:**
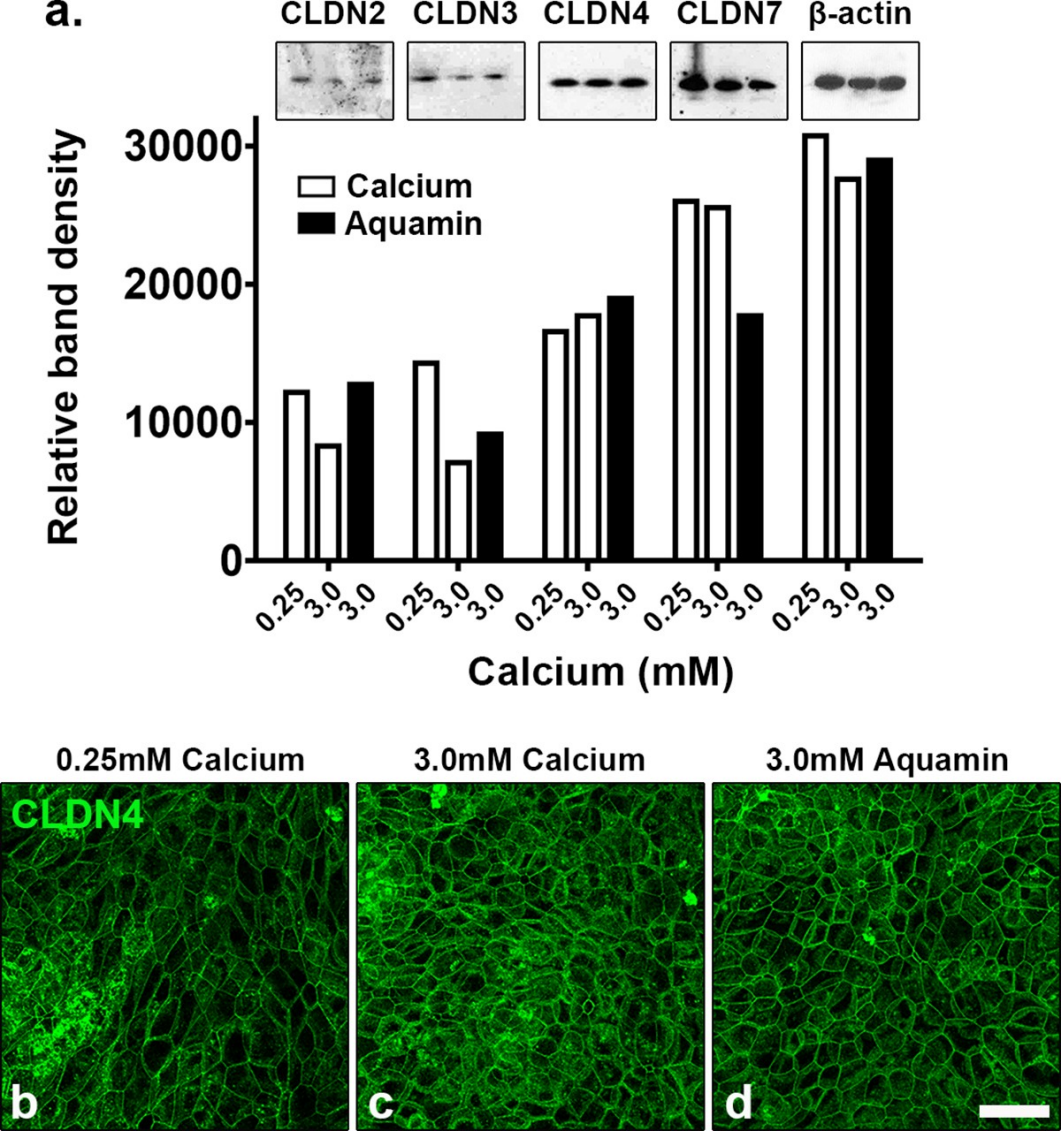
Evaluation of claudin expression by western blotting and confocal fluorescence microscopy. Colonoids were plated on transwell membranes and incubated under the indicated conditions. After TEER assessment at day-5, lysates from some membranes were prepared for western blotting and additional membranes were prepared for confocal fluorescence microscopy. **a**: Western blot: 10 μg of protein from each condition was used. β-actin was used as a loading control. Band quantitation was done using ImageJ software. **b-d**: Claudin-4 (max projected) was assessed by confocal fluorescence microscopy. Scale bar = 10 μm. CLDN = claudin.

Fig 3 shows expression of claudin-2, -3, -4 and -7 by Western blotting (Fig 3A) and claudin-4 by immunofluorescence (Fig 3B–3D). Consistent with occludin results presented above, there was little change in any of the four tight-junction proteins resulting from intervention with either calcium alone or Aquamin.

**Fig 4:**
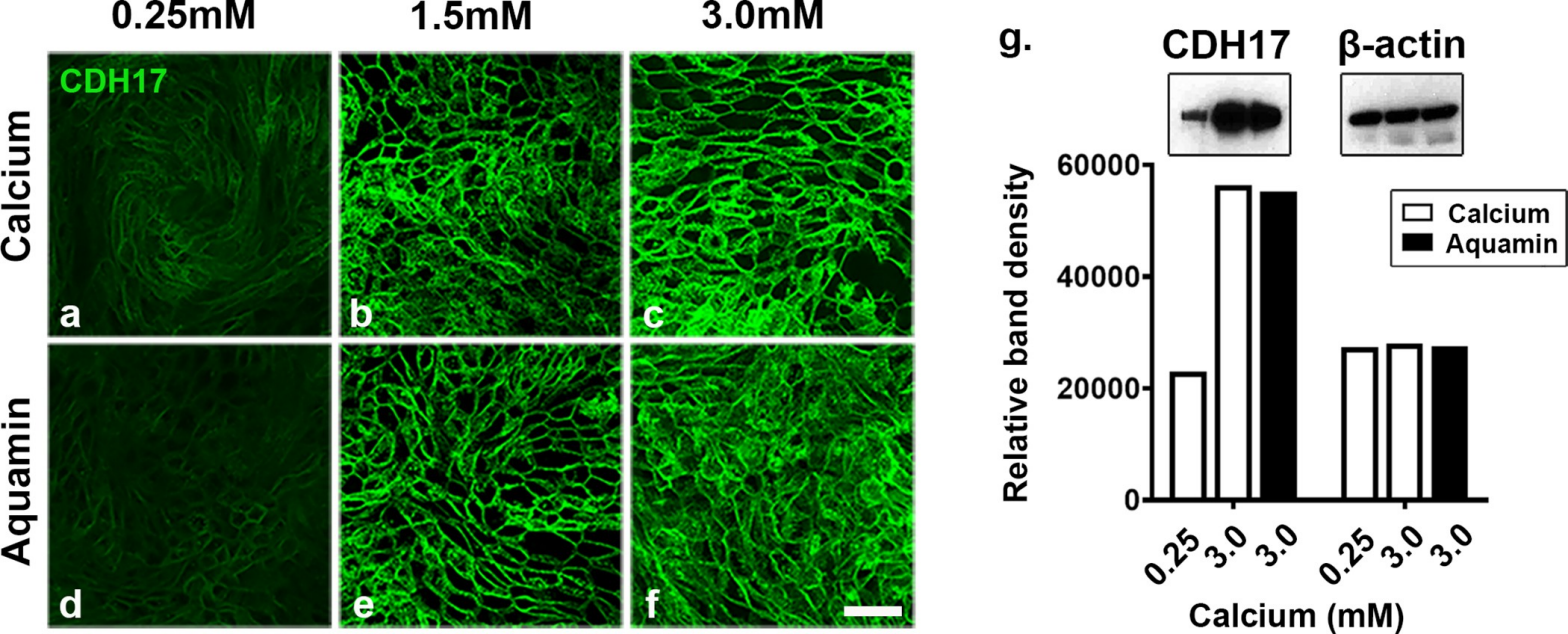
Evaluation of cadherin-17 expression by confocal fluorescent microscopy and western blotting. Colonoids were plated on transwell membranes and treated as indicated. **a-f**: Confocal fluorescence microscopy. After TEER assessment at day-5, membranes were prepared and stained for cadherin-17 expression (max projected). Scale bar = 10 μm. **g**: Protein isolated from each condition was assessed for cadherin-17 expression by western blotting. 10 μg of protein from each condition was used. β-actin was used as a loading control. Band quantitation was done using ImageJ software. CDH17 = cadherin-17.

Using a separate transwell membrane, colonoid cells (day-5) were stained with an antibody to cadherin-17. Results shown in Fig 4 (panels a-f) indicate low-level cadherin-17 expression in control and a dramatic up-regulation following supplementation with calcium alone (1.5 or 3.0 mM) or with Aquamin providing the same amount of calcium. Of interest, and unlike what was observed with desmoglein-2 at 0.25 mM calcium, cadherin-17 expression in the low-calcium environment was not patchy. Rather, the cell border was well-defined, even though only faintly visible. Western blot results shown in Fig 4G, confirm the low-level expression under control conditions and up-regulation with supplementation.

### Colonoid cohesion

**Fig 5:**
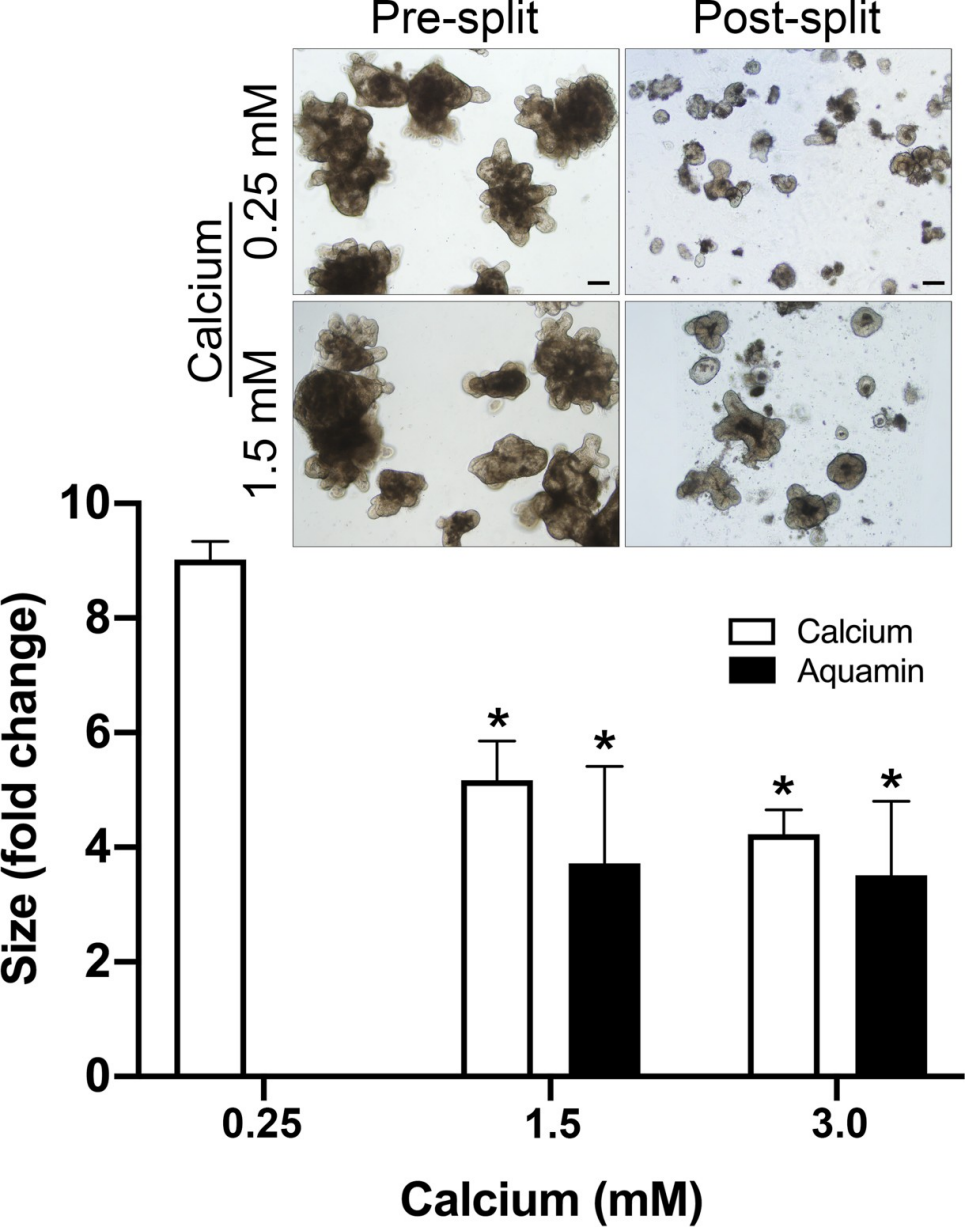
Colonoid cohesion. Colonoids were maintained for 14 days in culture under the indicated conditions. At the end of the incubation period, the size of multiple individual colonoids was assessed by measuring surface area in phase-contrast images. Following this, the colonoids were harvested and fragmented for subculture. After plating, the fragmented colonoids were again “sized.” Values shown represent the change in surface area means and standard deviations of individual colonoids based on three separate experiments representing colonoids from three different subjects with a minimum of 80 colonoids from each subject assessed per treatment group in both pre- and post-split cultures. Data were compared for statistical differences using ANOVA followed by unpaired-group comparisons. * indicates a difference from 0.25 mM calcium at *p*<0.05. Inset: Representative examples of pre-split and post-split colonoids. Scale bar = 200 μm.

Fig 5 presents results from colonoid cohesion studies. For these studies, colonoids were maintained for a 14-day period in a mix of L-WRN and KGM-Gold containing either 0.25 mM calcium (control) or the same medium supplemented with additional calcium alone or with Aquamin. At the end of the incubation period, surface area measurements were made on multiple individual colonoids as described in the Materials and Methods Section. Following this, subculture was done and individual colonoids were “sized” again in the same manner. With colonoids maintained in culture medium containing 0.25 mM calcium, there was an approximately 9-fold reduction in colonoid size during subculture (i.e., average post-split size of individual colonoids compared to average pre-split size). In comparison, colonoids incubated in culture medium containing 1.5 or 3.0 mM calcium demonstrated only 5.2-fold and 4.2-fold reductions in size. With Aquamin, pre-split versus post-split differences in colonoid size were even smaller (i.e., 3.7-fold and 3.5-fold reductions).

## Discussion

Calcium is the quintessential promoter of epithelial cell differentiation [19]. Calcium levels above approximately 1.0 mM are required for differentiation to occur with epithelial cells in monolayer culture. It was surprising, therefore, when our recent studies [20,21] demonstrated a substantial level of differentiation (indicated by colonoid gross and histological appearance and by differentiation marker [CK20] expression) in normal human colonoids maintained under low-calcium (0.25 mM) conditions. In these colonoids, the tight junctional protein, occludin, was detectable at the cell surface by immunostaining and tight junctions were evident by electron microscopy. In parallel, proteomic analysis showed that a number of tight junction proteins (including occludin) were detected, but only minimally up-regulated with calcium supplementation as compared to control. In contrast, however, a number of other cell surface and extracellular matrix proteins were minimally expressed under low-calcium conditions, but substantially up-regulated with calcium supplementation. Among these were desmosomal proteins (desmoglein-2, desmocollin-2 and desmoplakin), several cadherin family members (cadherin-17, protocadherin-1, cadherin-related family members-2 and -5), and proteins found at the apical surface of the colonic epithelium (mucins, trefoils and CEACAMs). Additionally, the major non-collagenous components of the basement membrane (laminin α, β and γ chains, nidogen and heparin sulfate proteoglycan) were also strongly up-regulated in response to calcium supplementation. The significance of these calcium concentration-related differences in protein expression to functional behavior was not addressed. In the present study, we used electrical resistance across colonoid-derived epithelial cells in 2-dimensional transwell membrane cultures as a measure of barrier integrity and permeability. In parallel, an assay for tissue cohesion in intact 3-dimensional colonoids was employed. Functional responses were compared with protein expression data.

Electrical resistance across the polarized gastrointestinal epithelial layer correlates with the existence of a barrier to small molecule passage between adjacent epithelial cells [2–4]; it is generally accepted that tight junctions, primarily, mediate this property [23]. Consistent with tight junction protein expression data—both in the intact colonoids [21] and cells derived from the colonoids (this report)—trans-epithelial electrical resistance was substantial under low-calcium conditions. In spite of the high background level, calcium supplementation increased TEER still further. This occurred without a major change in occludin expression or in the expression pattern of several claudins. Taken together, these data show that the low calcium level needed to initiate differentiation in colonoid culture is also sufficient to support barrier formation but does not result in maximal barrier expression.

Formation of tight junctions between adjacent epithelial cells follows cell-cell adhesion and formation of adherens junctions. Cadherin-1 (E-cadherin) is primarily responsible for this initial adhesive interaction [32,33]. This is of interest because although several cadherin family members were induced with calcium supplementation, cadherin 1 was not one of them. Rather, cadherin-1 was highly expressed in the low-calcium environment [21,34] and not further induced with calcium supplementation.

While trans-epithelial electrical resistance was generated under low-calcium conditions, the same low calcium level did not support strong tissue cohesion. Rather, a substantial increase in cohesion was observed with either calcium alone or Aquamin at 1.5 and 3.0 mM as compared to the low-calcium control. Increased cohesion correlated with the up-regulation of desmosomal proteins (citation 21 and this report) and with an increase in actual desmosomes seen at the ultrastructural level [21]. Of interest, increased cadherin-17 expression occurred concomitantly with elevated expression of desmoglein-2 While cadherin-17 does not participate in the formation of adherens junctions, this cadherin is highly responsive to small changes in calcium concentration and does contribute to homotypic and heterotypic cell adhesion in the intestinal epithelium [35]. Cadherin-17 up-regulation with calcium supplementation in parallel with desmosomal protein up-regulation could help organize desmosomal proteins into the structures that are responsible for strong cell-cell cohesion.

While the relationship between desmosome formation and tissue cohesion is clear, the role these structures play in the regulation of small-molecule permeability is less so. Perhaps the increase in electrical resistance seen with calcium supplementation is a reflection of a direct contribution of desmosomes to the permeability barrier. A recent study demonstrated a signaling role for desmoglein-2 in tight junction formation during barrier repair [31]. Alternatively, the desmosome contribution may be indirect. It is difficult to envision precise control of permeability in the absence of strong tissue cohesion–especially in a mechanically-active tissue such as the colon. Additional studies will be needed to address this complex issue.

Along with molecules that directly affect cell-cell adhesion, our recent study [21] also demonstrated an up-regulation of several other moieties that may contribute to overall barrier formation in the colon. Among these are components of the basement membrane (laminin α, β and γ chains, nidogen and heparan sulfate proteoglycan). In a simple epithelium such as the colon, there is only one layer of epithelial cells, and every cell resides on the basement membrane. If the basement membrane is not optimally constituted, or if cells cannot properly attach to the basement membrane, rapid apoptosis and sloughing of the cell layer will occur [36,37]–making more-subtle effects on barrier function moot. Furthermore, the basement membrane itself influences the transit of cells and macromolecules from the colonic fluid into the interstitium [14,15].

Finally, molecules that form the carbohydrate-rich layer above the colonic epithelium (mucins) were also found to be up-regulated with supplementation. These carbohydrate-rich molecules trap bacteria and other particulates, preventing them from reaching the epithelial cell surface in the first place [38,39]. All of these moieties, undoubtedly, contribute to an overall protective barrier in the colon.

There is a strong relationship between defective barrier function and inflammatory diseases in the gastrointestinal tract. Barrier dysfunction has been noted in both Crohn’s disease and ulcerative colitis [40–42] and is postulated as a component of irritable bowel syndrome [43] and celiac disease [44]. Defective barrier function and chronic inflammation have also been seen even without overt bowel disease; e.g., as a consequence of high-fat diets [45], hyperglycemia [46], psychological stress [47] and hypoxia [48]. It is well-accepted that inflammation can damage the barrier [49]. Once barrier defects occur, bacteria, bacterial products and other toxins / allergens can gain access to the interstitium. This promotes additional inflammation, which, in turn, leads to additional barrier breakdown. Our findings in no way contradict this understanding. They argue, however, that even in the presence of conditions that promote barrier damage in the gastrointestinal tract, having a level of calcium intake that supports optimal elaboration and function of key barrier proteins would be beneficial. Our data also argue that focusing only on tight junctions and small molecule permeability may be too limited to fully appreciate barrier changes in colonic disease.

In this study, calcium provided as a single agent was compared to calcium delivered as part of a calcium- and magnesium-rich multi-mineral product. In both the TEER and cohesion assays, responses to the multi-mineral intervention were greater than those to calcium alone. What accounts for the enhanced response to the multi-mineral intervention is not known with certainty, but previous studies provide insight. Aquamin itself, as well as certain of the individual trace elements present in the multi-mineral product, activate the extracellular calcium-sensing receptor, a critical target in epithelial cell responses to calcium, more effectively than calcium itself [50–52]. In the presence of the additional trace elements, a “left-shift” in calcium response occurs. Additional studies have demonstrated that trace elements of the lanthanide family (present in Aquamin) have potent effects on calcium channels [53] as well as effects on calcium “pumps” [54]. Effects on one or more of these critical effectors of calcium function could interfere with calcium responses. Alternatively, rather than modulating calcium signaling, *per se*, the major effect of the multi-mineral supplement may be directly on the adhesion process. While extracellular calcium is critical to both cell-cell and cell-matrix adhesion, other divalent cations (notably, magnesium and manganese) are also required for optimal adhesive interactions [55,56]. These possible mechanisms are not mutually exclusive.

## Conclusions

The findings presented here may help explain how an adequate daily intake of calcium contributes to health, despite there being little change in colonic histological features over a wide-range of calcium intake amounts [57–60]. While having an adequate daily intake of calcium is important to health, most individuals are calcium deficient. Studies from North America, Europe and Australia have all shown that a majority of individuals do not achieve a minimal daily calcium intake [61–66]. The “Western-style diet” has been thought to underlie chronic calcium deficiency, but a recent study showed that even where a rural agrarian diet is the norm, significant calcium-deficiency exists [67]. From a public health standpoint, ensuring an adequate calcium-intake along with cofactors such as vitamin D and, perhaps, additional trace elements needed for optimal calcium uptake and function may provide a cost-effective way to improve overall health and well-being in a large segment of the population. It might be noted in this regard that in addition to the consequences of epithelial barrier dysfunction in the gastrointestinal tract, a relationship between epithelial barrier dysfunction in lung and skin has been suggested for both asthma and eczema [68,69].

A critical issue is whether and to what extent these findings in colonoid culture are reflective of what occurs *in vivo* in the intact colon. Can we, in fact, duplicate the colonoid culture findings with intervention in human subjects? We are currently in the midst of a 90-day interventional trial in healthy adult human subjects, comparing Aquamin (delivering 800 mg of calcium per day) to calcium alone (800 mg/day) and to placebo for effects on the same colon biomarkers of growth, differentiation and barrier formation as assessed in colonoid culture (clintrials.gov; NCT02647671). Results of the ongoing trial should be instructive as to the relative efficacy of the multi-mineral approach compared to calcium alone as well as provide an indication of tolerability and safety. Initial data generated in the ongoing interventional trial indicate that safety and tolerability are unlikely to be problems. Additionally, changes in gut microbial population and attendant metabolic features were seen in the same study [70]. Data from the interventional trial, along with those data generated here and in our recent colonoid studies [20,21], will, ultimately, demonstrate i) whether multi-mineral supplementation has efficacy, ii) whether efficacy with the multi-mineral approach is greater than can be achieved with calcium alone and iii) whether the presence of multiple trace elements can provide efficacy at lower calcium dose than needed with calcium alone.

## Supporting information

S1 Table Mineral composition of Aquamin^®^. (PDF)

S2 Table Antibody characteristics. (PDF)

S1 File Source documentation and raw blots for western blotting data in Figs 2, 3 and 4. (PDF)

S2 File (PDF)

S1 Movie A confocal Z-stacks generated movie. (AVI)
