# Supplementary material for: Differentiation of human colon tissue in culture: Effects of calcium on trans-epithelial electrical resistance and tissue cohesive properties
Source: PLoS One. 2020 Mar 5;15(3):e0222058. doi: 10.1371/journal.pone.0222058 (PMC7058309; doi:10.1371/journal.pone.0222058)
Supplement: S1 Table — (PDF) [file pone.0222058.s001.pdf]

S1 Table. Aquamin® Mineral Analysis.

Element	μg/g	Element	μg/g	Element	μg/g
Aluminum	21.6	Hafnium	0.038	Rubidium	0.031
Antimony	0.69	Holmium	0.010	Ruthenium	0.137
Arsenic	0.239	Indium	<0.001	Samarium	0.037
Barium	1.76	Iodine	1.81	Scandium	0.469
Beryllium	<0.5	Iridium	<0.001	Selenium	<0.5
Bismuth	<0.5	Iron	143	Silicon	16.8
Boron	13.7	Lanthanum	<0.5	Silver	<0.5
Cadmium	0.220	Lead	0.084	Sodium	2,206
Calcium	117,000	Lithium	<0.5	Strontium	882
Carbon	26,600	Lutetium	<0.001	Sulfur	1,241
Cerium	0.314	Magnesium	10,210	Tantalum	0.043
Cesium	0.001	Manganese	25.4	Tellurium	<0.5
Chloride	612	Mercury	<0.001	Terbium	0.007
Chromium	<0.5	Molybdenum	<0.5	Thallium	<0.5
Cobalt	<0.5	Neodymium	0.170	Thorium	1.30
Copper	<0.5	Nickel	0.75	Thulium	0.004
Dysprosium	0.045	Niobium	<0.5	Tin	0.029
Erbium	0.033	Osmium	<0.001	Titanium	11.4
Europium	0.013	Palladium	0.179	Tungsten	<0.5
Fluoride	3.57	Phosphorous	189	Vanadium	<0.5
Gadolinium	0.044	Platinum	<0.001	Ytterbium	0.030
Gallium	0.307	Potassium	70.0	Yttrium	<0.5
Germanium	<0.001	Praseodymium	0.040	Zinc	6.07
Gold	<0.5	Rhenium	0.001	Zirconium	<0.5
		Rhodium	0.061		

Source: 2017 Test Certificate for Aquamin® Soluble, by Advanced Laboratories, Inc. (Salt Lake City), for client Marigot Limited (Ireland).
The levels of individual trace elements were determined by Inductively Coupled Plasma Optical Emission Spectrometry (ICP-OES) except Carbon (determined by LECO), Chloride, Iodine (determined by Titration), and Fluoride (determined by AOAC 939.11).
