# Supplementary material for: Differentiation of human colon tissue in culture: Effects of calcium on trans-epithelial electrical resistance and tissue cohesive properties
Source: PLoS One. 2020 Mar 5;15(3):e0222058. doi: 10.1371/journal.pone.0222058 (PMC7058309; doi:10.1371/journal.pone.0222058)
Supplement: S2 Table — (PDF) [file pone.0222058.s002.pdf]

S2 Table. Antibody characteristics

A. Confocal Microscopy Antibodies

Antibody	Company	Catalog #	Fluorochrome	Staining Dilution
Cadherin-17	Novus Biologicals	NBP2-12065AF488	Alexa Fluor 488	1:200
Claudin-4	Invitrogen	329488	Alexa Fluor 488	1:400
Desmoglein-2	eBioscience	53-9159-80	Alexa Fluor 488	1:200
Occludin	Invitrogen	331594	Alexa Fluor 594	1:400

B. Western Blotting Antibodies

Antibody	Company	Catalog #	Concentration	Secondary Ab
Cadherin-17	abcam	Ab109190	0.166 μg/ml	Invitrogen mouse anti-rabbit, HRP (cat#31464)
Claudin-2	Invitrogen	32-5600	1.0 μg/ml	Cell Signaling horse anti-mouse, HRP (7076S)
Claudin-3	Invitrogen	34-1700	1.0 μg/ml	Invitrogen mouse anti-rabbit, HRP (cat#31464)
Claudin-4	Invitrogen	32-9400	1.0 μg/ml	Cell Signaling horse anti-mouse, HRP (7076S)
Claudin-7	Invitrogen	37-4800	1.0 μg/ml	Cell Signaling horse anti-mouse, HRP (7076S)
Desmoglein-2	Invitrogen	MA1-91589	1.0 μg/ml	Cell Signaling horse anti-mouse, HRP (7076S)
Occludin	Invitrogen	PA5-20755	1.0 μg/ml	Invitrogen mouse anti-rabbit, HRP (cat#31464)
Beta-Actin	Cell Signaling	5125S	1:1000	Invitrogen mouse anti-rabbit, HRP (cat#31464)
