# Supplementary material for: Differentiation of human colon tissue in culture: Effects of calcium on trans-epithelial electrical resistance and tissue cohesive properties
Source: PLoS One. 2020 Mar 5;15(3):e0222058. doi: 10.1371/journal.pone.0222058 (PMC7058309; doi:10.1371/journal.pone.0222058)
Supplement: S1 File — (PDF) [file pone.0222058.s003.pdf]

S1 File: Source documentation for western blot data in Figures 2, 3 and 4: A total of 8 proteins were probed by western blotting and presented in the manuscript. These were desmoglein-2, occludin, cadherin-17, claudin-2, claudin-3, claudin-4 and claudin-7 along with β-actin as a control.

Procedure: Colonoid cell lysates were prepared and separated by gel electrophoresis using pre-cast gels. Following this, the proteins were transferred to nitrocellulose sheets in the standard manner. A total of three electrophoresis runs were used to acquire the needed images. There were three conditions (lanes) for each experiment. These are presented in the final composite figure as lane 1: 0.25 mM calcium (control); lane 2: 3.0 mM calcium and lane 3: Aquamin providing 3.0 mM calcium. Following completion of the electrophoresis and protein transfer, the appropriate antibodies were used to probe for the proteins of interest. Images were then obtained on films as described in the Materials and Methods Section of the manuscript. Each of the three nitrocellulose sheets along with the films generated from each nitrocellulose sheets are shown together. They are labeled as #1, #2 and #3 for the supplement file.

Nitrocellulose sheet #1 (and accompanying films). This nitrocellulose sheet was cut horizontally and separate pieces were used to probe for occludin and desmoglein-2. β-actin was probed as control. Desmoglein-2 is shown at the top of the film. There is little detectable signal in the control lane (0.25 mM calcium) but a strong and equivalent signal in the other two lanes (3.0 mM calcium and Aquamin). Occludin (shown in the middle) demonstrates comparable signal under all three conditions. β-actin shown in the lower part of the film was also probed. It appears to be comparable in all three lanes though well over-exposed. [Note: the nitrocellulose film was cut into 4 parts because the last part was probed for cadherin-17. Unfortunately, there was absolutely no signal with this protein and we subsequently obtained a different antibody in order to redo the analysis].

Nitrocellulose sheet #2 (and accompanying film). This nitrocellulose sheet was cut into multiple sections and used for multiple different analyses (some unrelated to the present study). Cadherin-17 is shown in the top-left portion of the film. The two dense bands correspond to calcium at 3.0 mM and Aquamin providing 3.0 mM calcium. The third, much thinner, band corresponds to the control (0.25 mM calcium). Shown below the cadherin-17 bands is the portion of the nitrocellulose film probed for β-actin (over exposed). The remainder of the film was used for purposes unrelated to this project.

Nitrocellulose sheet #3 (and accompanying films). For this run, the acrylamide gel was loaded with protein from the same three conditions (as above) in triplicate. Then, after transfer to nitrocellulose, the sheet was cut vertically with each piece containing protein from each of the three conditions. We used this nitrocellulose sheet to probe for claudin-2, claudin-3, claudin-4 and claudin-7. Since all of these moieties are similar in molecular weight, we could not cut the sheet horizontally to any advantage. In the film immediately to the right of the nitrocellulose sheet, one can see a weak (barely detectable, but equivalent) signal for claudin-2 (right side), a very over-exposed signal for claudin4 (middle) and a strong (but equivalent) signal for claudin-7 (left side). The lower portions of the film are unrelated to the current project. For the next film in the series, the portion of the nitrocellulose sheet with claudin-4 was removed and the signal

from the other two portions of the nitrocellulose sheet generated. Again one can see a weak but equivalent signal for claudin-2 and a strong, equivalent signal for claudin-7. Then, the portion of the nitrocellulose film probed for claudin-4 was used to generate a signal for claudin-4 using a "touch prep". This produced the strong and equivalent signal in all three lanes. Following this, a portion of the nitrocellulose sheet was washed and then re-probed with antibody to claudin-3 (gels in the lower portion of the file). After development, a signal for claudin-3 was detected. Of the two gels presented, the right-hand gel was used for the figure shown in the manuscript and in the composite picture presented here. In this film β-actin can also be seen.

Note: In the actual gels, lanes 1, 2 and 3 go from left to right. The films are mirror images so it appears that lanes 1, 2 and 3 go from right to left. When the final figures were generated, the lanes were presented as run in the gels.

Composite image. This file shows the lanes that were used to generate the "presentable" images in the body of the manuscript.
(A). A montage of all the original Western blots (to assess the expression of Claudin-2, -3, -4, -7 and Cadherin-17 in the colonoid-derived monolayer) is presented. A Western blot including the β-actin loading control is also shown. Arrow represents the actual bands. Claudins have a molecular weight of ~20kDa. (B) and (C). Occludin (OCLN) and Desmoglein-2 (DSG2) protein expression. These two were run on the same membrane and incubated with corresponding primary antibodies after cutting the membrane horizontally. Occludin has a molecular weight of ~60kDa. The predicted band location for Cadherin-17 and Desmoglein-2 is ~95kDa and ~125 kDa.

Nitrocellulose Membrane #1

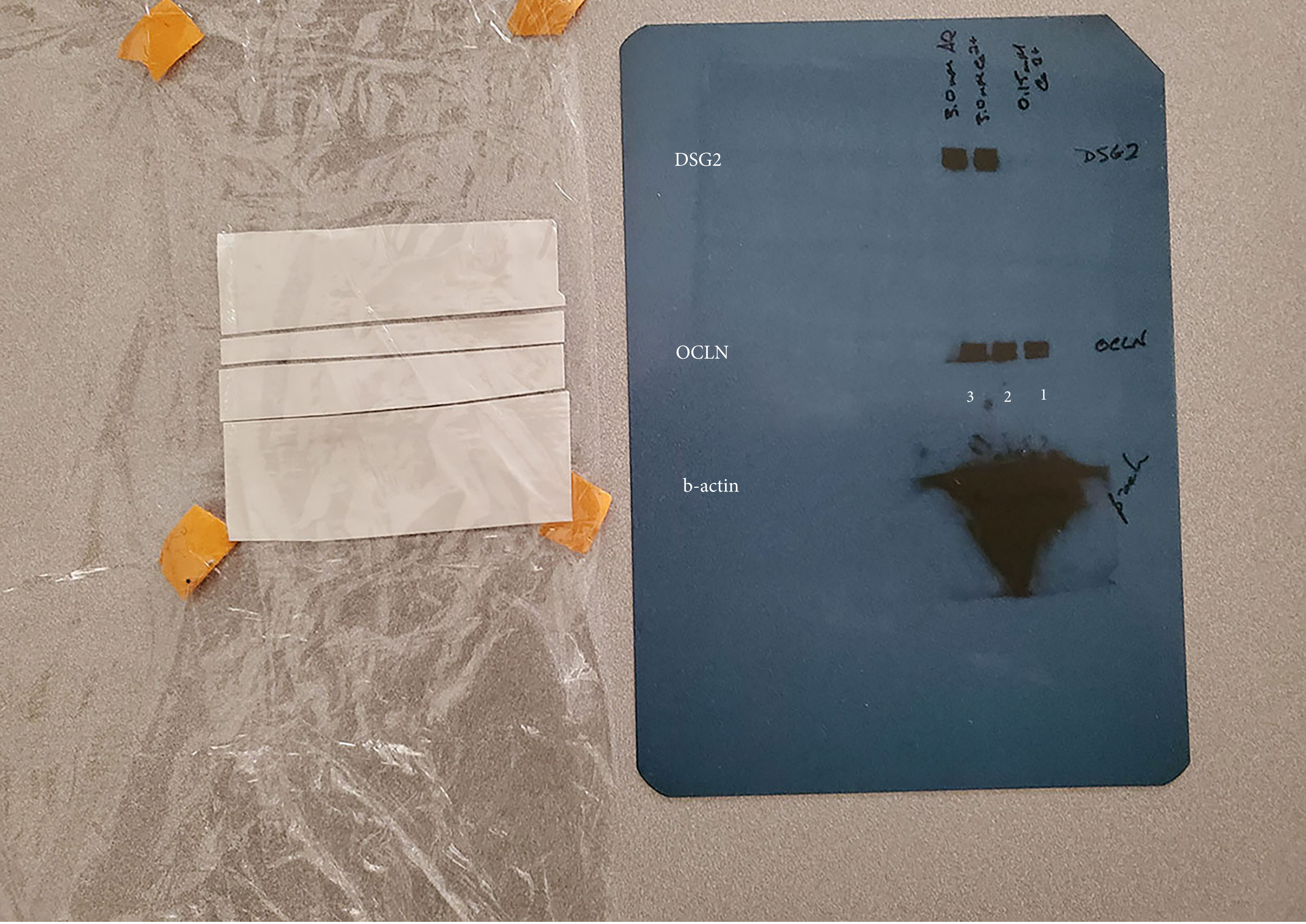

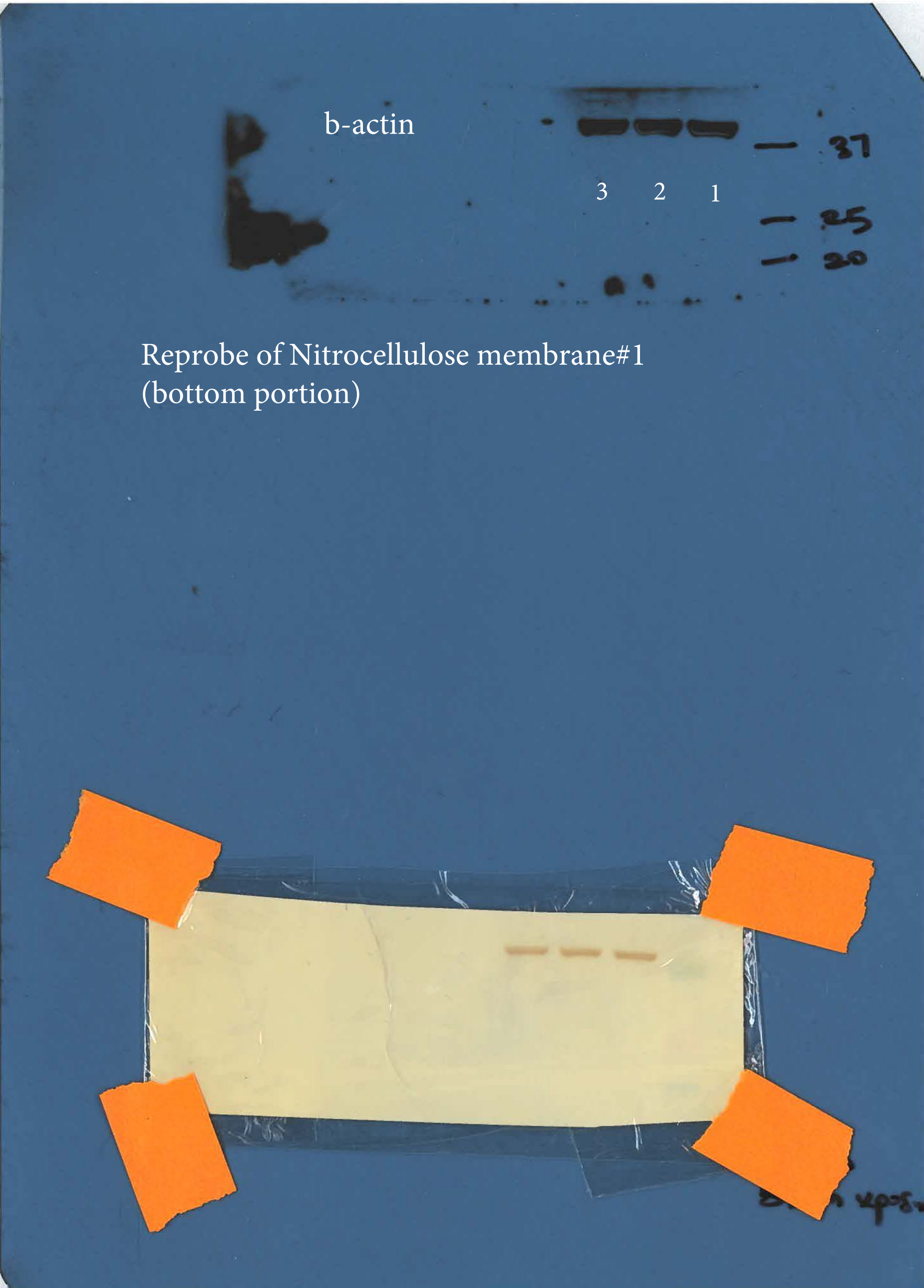
b-actin
3
2
1
37
25
20
Reprobe of Nitrocellulose membrane#1
(bottom portion)

Nitrocellulose Membrane #2

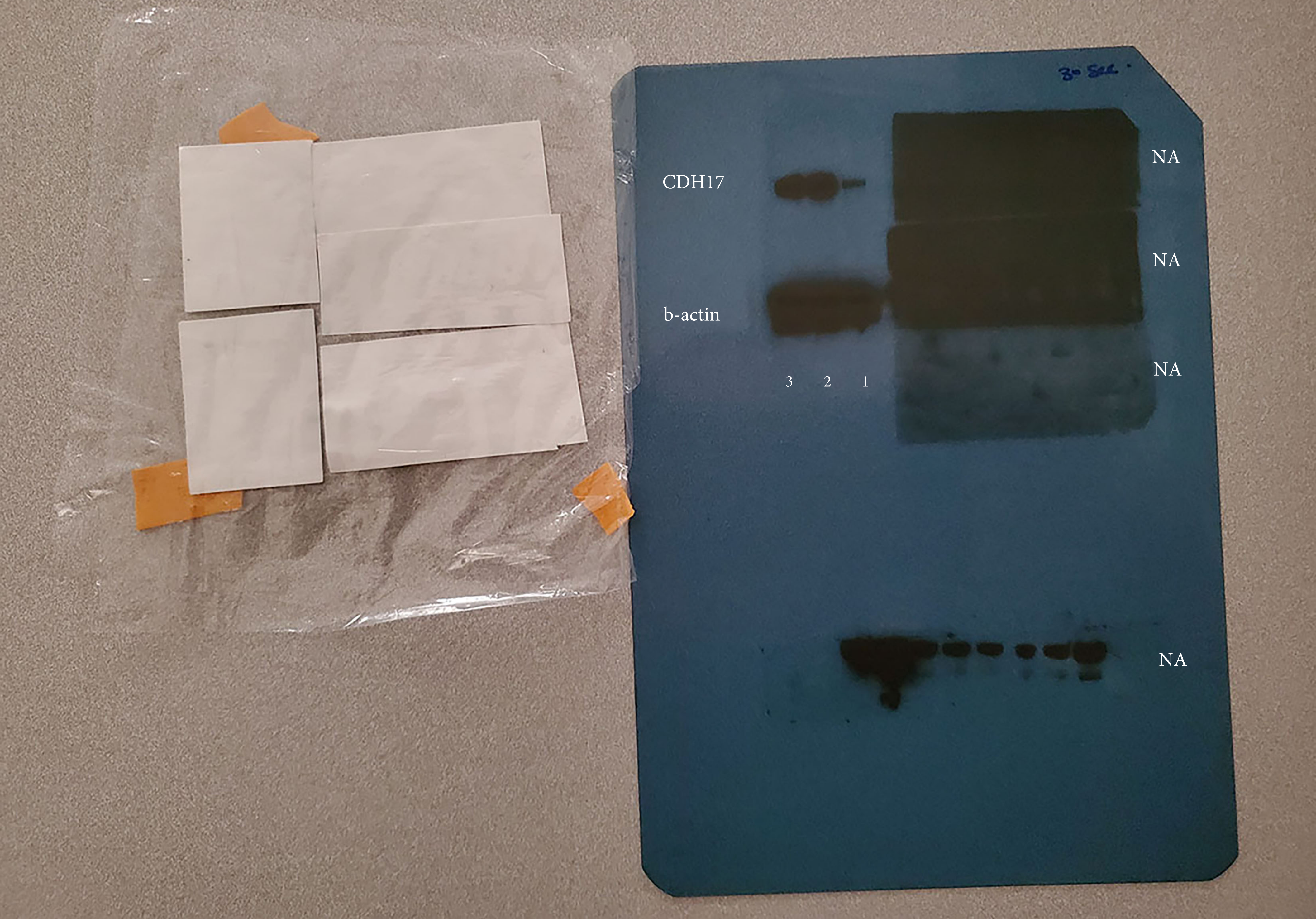

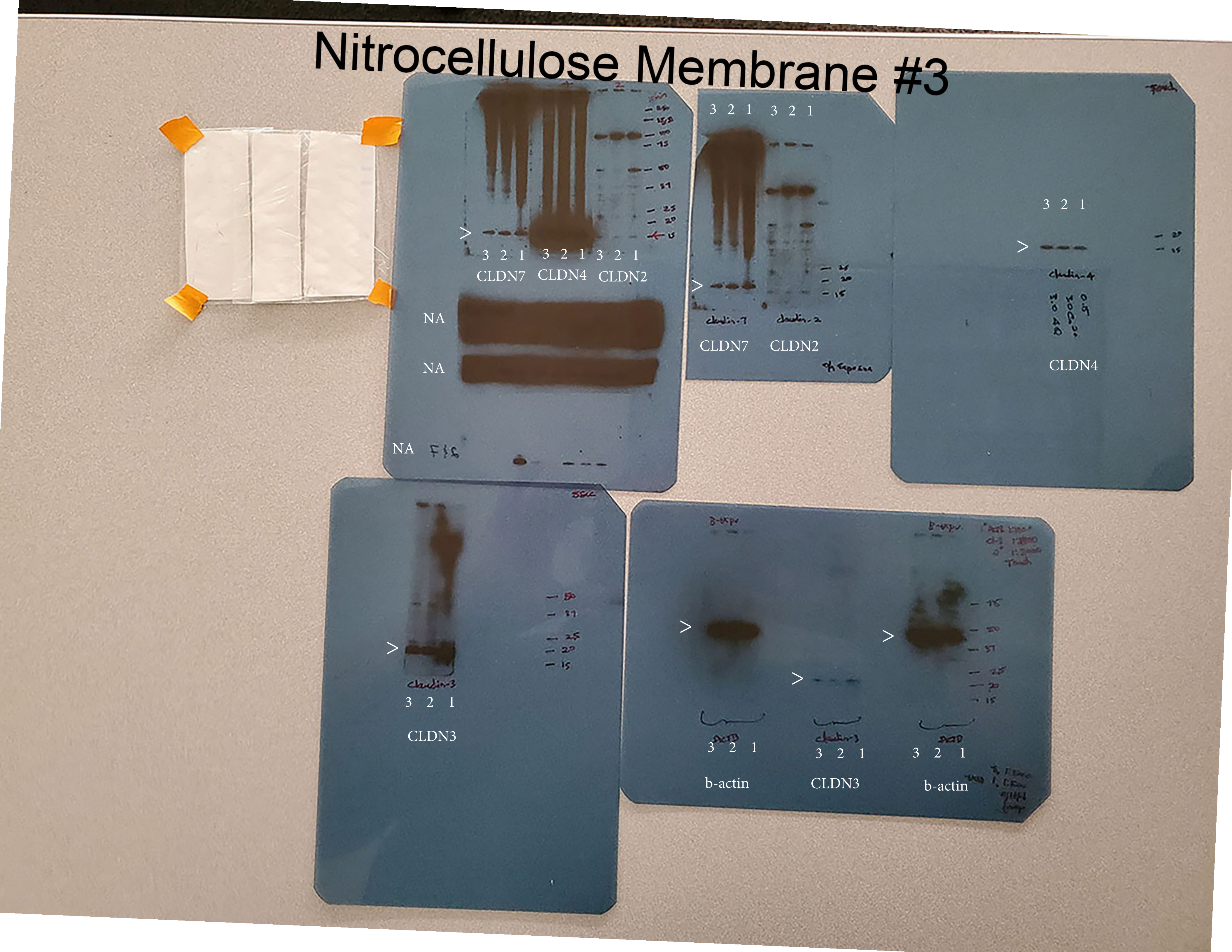
Nitrocellulose Membrane #3
3 2 1 3 2 1 3 2 1
CLDN7 CLDN4 CLDN2
NA
NA
NA
3 2 1 3 2 1
CLDN7 CLDN2
3 2 1
CLDN4
3 2 1
CLDN3
3 2 1 3 2 1 3 2 1
b-actin CLDN3 b-actin

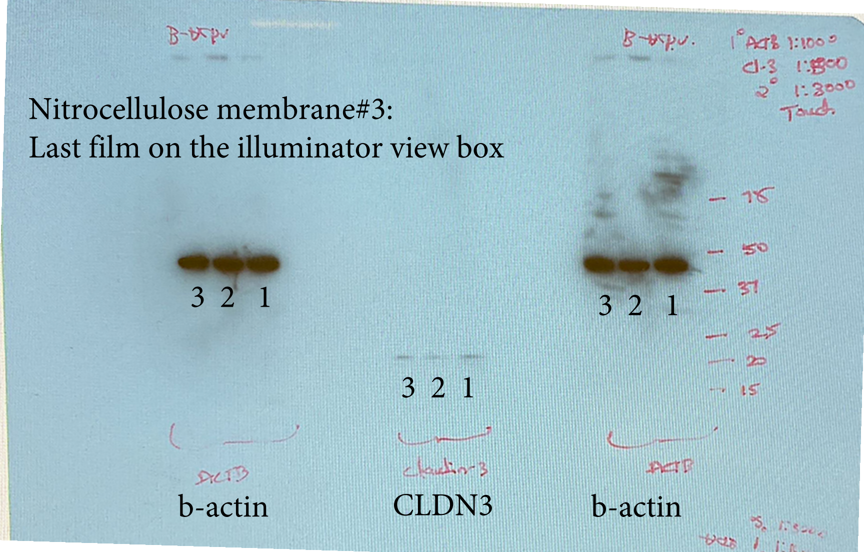
Nitrocellulose membrane#3:
Last film on the illuminator view box
3 2 1
3 2 1
3 2 1
75
50
37
25
20
15
b-actin
CLDN3
b-actin

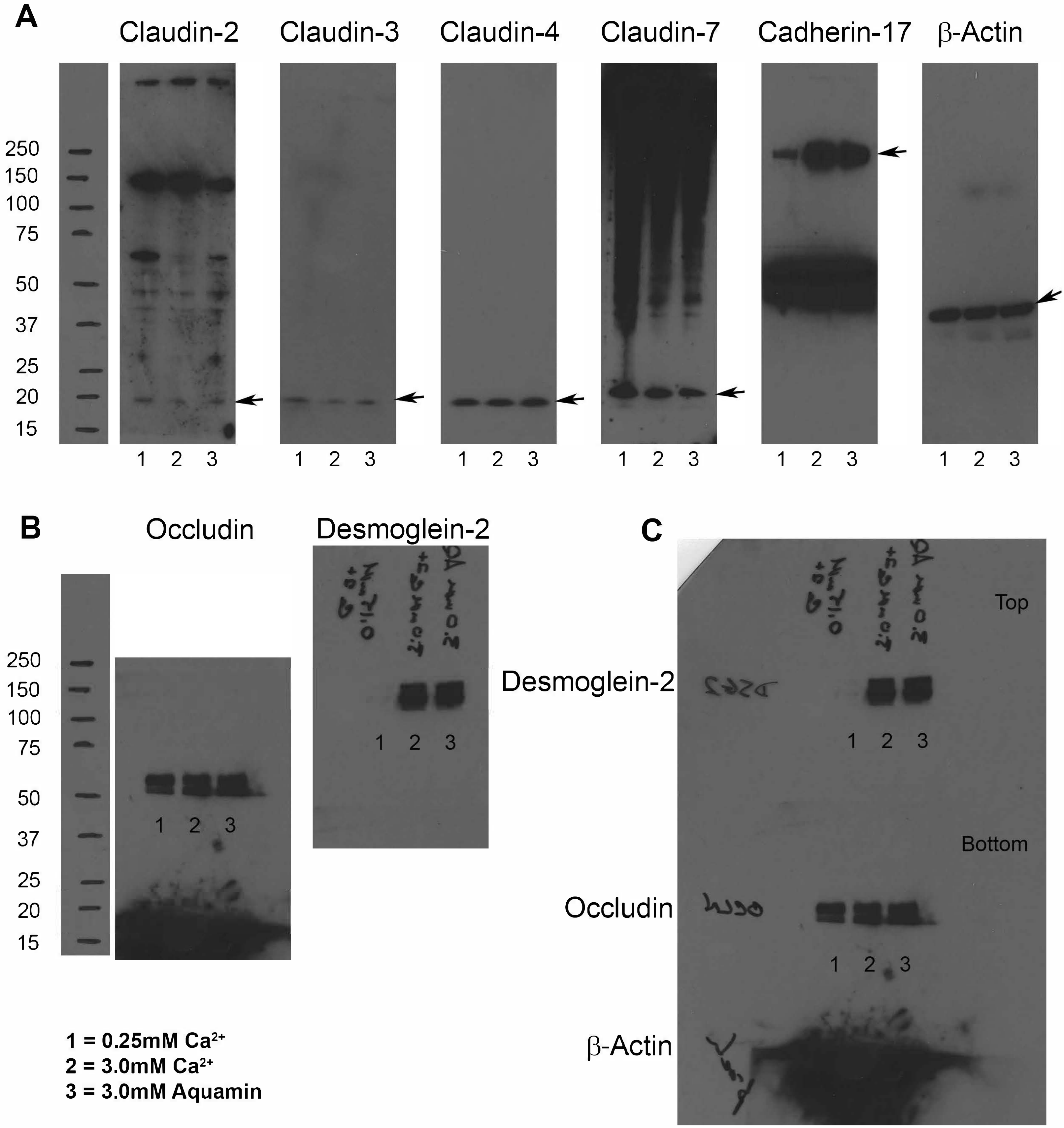

note OCLN and DSG2 were probed on the same membrane, that was cut horizontally (into top and bottom membranes) prior to primary antibody staining and developed as shown in C.
