# Supplementary material for: Differentiation of human colon tissue in culture: Effects of calcium on trans-epithelial electrical resistance and tissue cohesive properties
Source: PLoS One. 2020 Mar 5;15(3):e0222058. doi: 10.1371/journal.pone.0222058 (PMC7058309; doi:10.1371/journal.pone.0222058)
Supplement: S2 File — (PDF) [file pone.0222058.s004.pdf]

Supplemental Information.

Supplemental material includes two tables, one file and one QuickTime movie.

S1 Table. Mineral composition of Aquamin®

S2 Table. Antibody Characteristics

S1 File. Source documentation and raw blots for western blotting data in Figures 2, 3 and 4.

S1 Movie. A confocal Z-stacks generated movie.

Movie S2 Legend. A 3D rotating representation of occludin and desmoglein-2 staining of colonoid derived-epithelial monolayer; a treatment response to high-calcium (Calcium 1.5mM). Confocal generated Z-stacks were rendered as a 3D movie using Fiji (ImageJ1.52n with Bio-Formats Importer plugin). A QuickTime movie (S1 Movie) from confocal generated Z-stacks (from high-calcium) highlights the fact that occludin (red) staining is apical and desmoglein-2 (green) staining starts apically and extends laterally covering the entire length of cells. This can be correlated with the data presented in Fig 2n.
